# Supplementary material for: Identification of the Interacting Domains Between Tissue Factor and β1-Integrin and the Signalling Properties of the Two Fibronectin-like Domains of Tissue Factor
Source: Cancers (Basel). 2025 Feb 14;17(4):644. doi: 10.3390/cancers17040644 (PMC11853675; doi:10.3390/cancers17040644)
Supplement: Supplementary file 1 [file cancers-17-00644-s001.zip › cancers-3405365-supplementary.pdf]

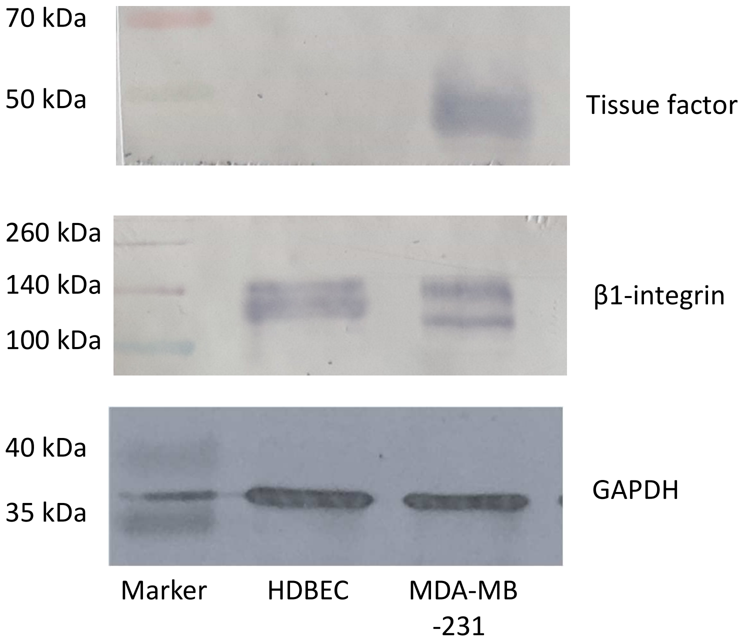

Figure S1. Assessment of TF and β1-integrin expression by Western blot. MDA-MB-231 breast cancer cells (3 × 10^5) and primary human dermal blood endothelial cells (HDBEC) (3 × 10^5) were lysed with Laemmli buffer and western blot analysis was carried out using a polyclonal rabbit anti-TF antibody, a polyclonal rabbit anti-β1-integrin antibody or a goat anti-GAPDH polyclonal antibody. Membranes were then probed with a goat anti-rabbit alkaline phosphatase-conjugated antibody or a donkey anti-goat alkaline phosphatase-conjugated antibody and bands were visualised using the Western Blue stabilised alkaline phosphatase-substrate and photographed. Images represent 2 separate experiments.

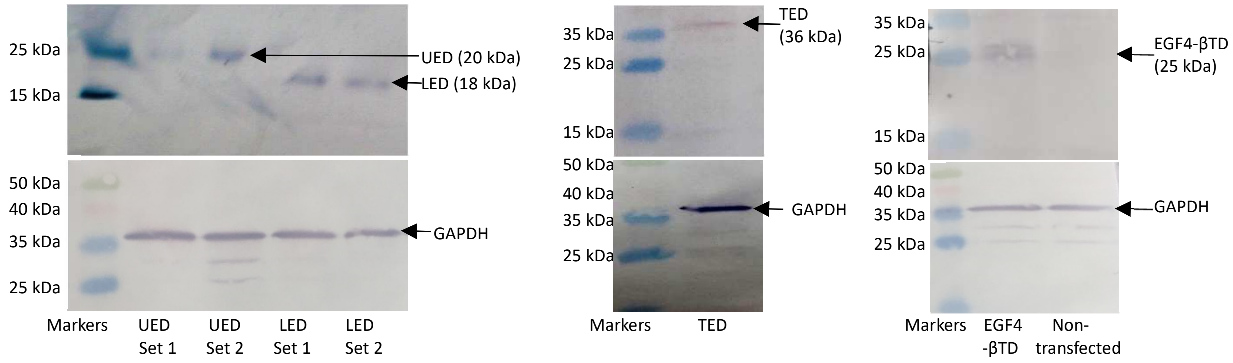

Figure S2. Verification of protein construct expression by Western blot. MDA-MB-231 cells (5×10^5) were transfected to express TED, LED, UED or EGF4-βTD peptides or were used non-transfected. Cells were lysed with Laemmli buffer and western blot analysis was carried out using a rabbit anti-HA-tag antibody (C29F4) or a goat anti-GAPDH polyclonal antibody. Membranes were then probed with a goat anti-rabbit alkaline phosphatase-conjugated antibody or a donkey anti-goat alkaline phosphatase-conjugated antibody and bands were visualised using the Western Blue stabilised alkaline phosphatase-substrate and photographed. Images represent 2 separate experiments.

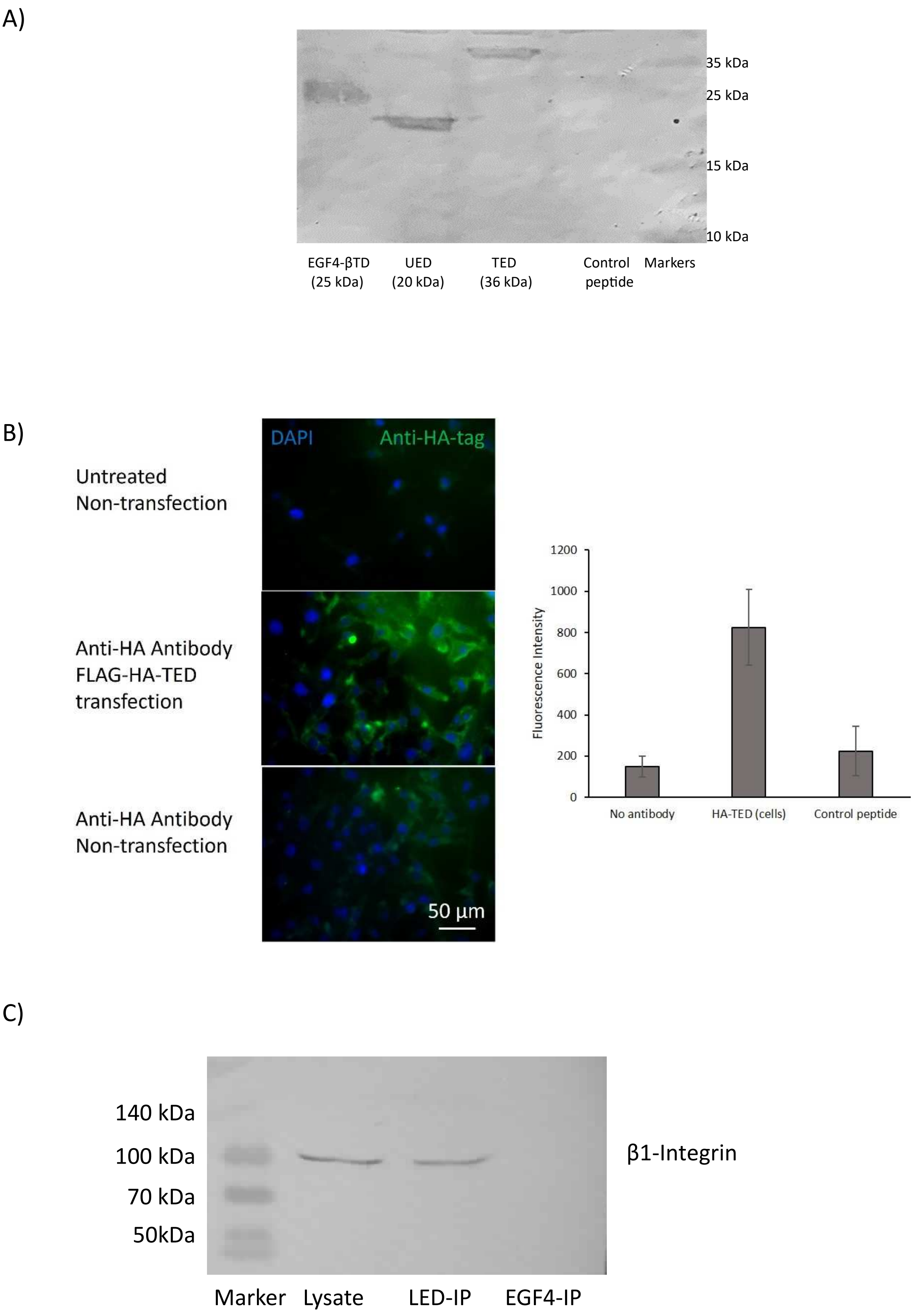

Figure S3. Verification of the externalisation of expressed protein constructs. (**a**) MDA-MB-231 cells (10^5) were transfected to express the EGF4-βTD, UED, TED constructs or empty vector used as control. The media from the cultures was collected and centrifuged to remove any cell debris. The supernatant was concentrated and

examined by western blot using rabbit anti-HA-tag (C29F4) antibody, developed with an alkaline phosphatase-conjugated goat anti-rabbit IgG antibody. The bands were visualised using Western blue stabilised alkaline phosphatase-substrate and photographed. Images represent 2 separate experiments. (**b**) MDA-MB-231 cells (10^4) were transfected to express the TED construct or empty vector used as control. Intact cells were probed with rabbit anti-HA-tag antibody (C29F4) and developed with NL493-conjugated anti-rabbit IgG antibody. The cells were examined by fluorescence microscopy at x40 magnification and the fluorescence intensities were quantified using ImageJ. (**c**) MDA-MB-231 cells (1.5×10^5) were transfected to express LED or EGF4 constructs for 48 h and were then lysed. The recombinant protein constructs were immunoprecipitated from the lysates using the anti-FLAG-tag (4 μg; C29F4) antibody and protein A-magnetic beads. Western blot analysis of the co-precipitated samples, along with input control samples of MDA-MB-231 and HBDEC cell lysate, was carried out using a polyclonal anti-β1-integrin antibody Bands were then visualised using the Western Blue stabilised alkaline phosphatase-substrate and photographed. Images represent 2 separate experiments.

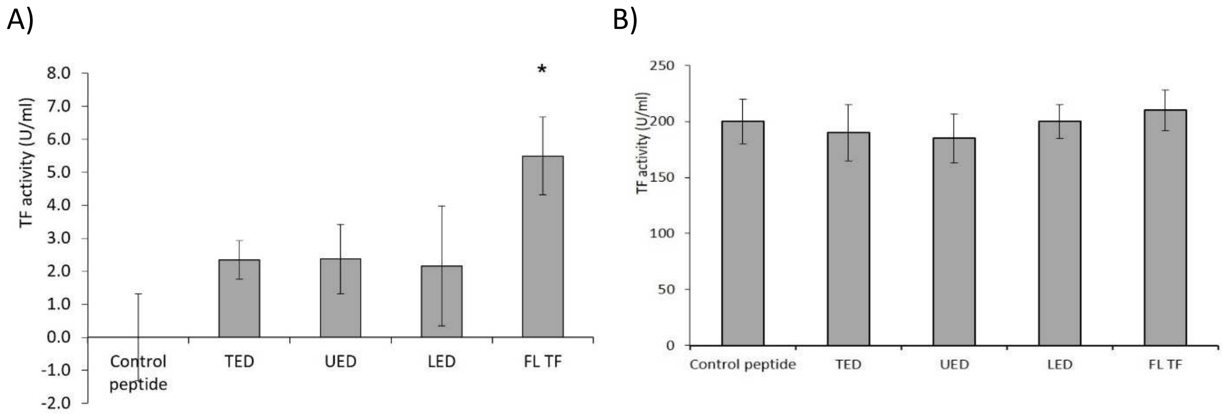

Figure S4. Quantification of TF activity on cell surface. (**a**) HDBEC and (**b**) MDA-MB-231 cells (10^4) were transfected to express the TED, LED or UED constructs or control peptide. Cells were incubated with 10 mM HEPES containing 2.4 nM fVIIa, 73.2 nM fX and 5 mM $CaCl_2$, for 2 h at 37°C. FXa chromogenic substrate (0.5 mg/ml) was then added and incubated at 37°C for a further 15 min. The absorption was measured at 405 nm using a microplate reader. TF activity was determined from a standard curve using diluted recombinant TF. (FL TF = Full-length TF).
